# Copper Kills *Escherichia coli* Persister Cells

**DOI:** 10.3390/antibiotics9080506

**Published:** 2020-08-12

**Authors:** Paula Maria Moreira Martins, Ting Gong, Alessandra A. de Souza, Thomas K. Wood

**Affiliations:** 1 Department of Chemical Engineering, Pennsylvania State University, University Park, PA 16802, USA; pmmm@outlook.com.br (P.M.M.M.); tinggong2017@hotmail.com (T.G.); 2 Biotechnology Lab, Centro de Citricultura Sylvio Moreira, Instituto Agronômico de Campinas, Cordeirópolis-SP 13490-970, Brazil; desouza@ccsm.br

**Keywords:** copper, persister, VBNC

## Abstract

Due to their reduced metabolism, persister cells can survive most antimicrobial treatments, which usually rely on corrupting active biochemical pathways. Therefore, molecules that kill bacterial persisters should function in a metabolism-independent manner. Some anti-persister compounds have been found previously, such as the DNA-crosslinkers mitomycin C and cisplatin, but more effective and lower cost alternatives are needed. Copper alloys have been used since ancient times due to their antimicrobial properties, and they are still used in agriculture to control plant bacterial diseases. By stopping transcription with rifampicin and by treating with ampicillin to remove non-persister cells, we created a population that consists solely of *Escherichia coli* persister cells. Using this population of persister cells, we demonstrate that cupric compounds kill *E. coli* persister cells. Hence, copper ions may be used in controlling the spread of important bacterial strains that withstand treatment with conventional antimicrobials by forming persister cells.

## 1. Introduction

Persister cells are a subpopulation of phenotypic variants that are tolerant to antimicrobial compounds. This phenotype was discovered in the early 1940s [1] and occurs primarily as a stress response [2,3,4]. Since they are metabolically inactive [5,6], antibiotics often do not eradicate these cells. The recurrence of microbial diseases has been attributed to persisters, so they constitute a reservoir of cells [7]. Notably, we found that for pathogenic and non-pathogenic *E. coli*, the viable but non-culturable state and persistence are equivalent [5].

Despite being refractory to many antimicrobial molecules, persister cells can be killed [8,9] by compounds such as cisplatin [10] and mitomycin C [11] that are FDA-approved for cancer treatments, and they can kill persister cells by crosslinking their genomic DNA while they “sleep”. These findings highlight new uses for well-known drugs. In addition, by screening compounds for activity directly on persister cells, the indigoid 5-nitro-3-phenyl-1*H*-indol-2-yl-methylamine hydrochloride was discovered that kills *E. coli*, *Pseudomonas aeruginosa*, and *Staphylococcus aureus* persister cells [12]. Additionally, retinoids have been found to kill *S. aureus* persisters [13], and other molecules have been identified [9], although they are not approved yet for persister treatment.

Copper compounds have been used for their antimicrobial properties since ancient times [14], and many different microorganisms are rapidly killed by copper ions [15,16]. Recently, copper alloys have been approved for use by the U.S. Environmental Protection Agency (Reg 82012-1 [17]), due to their effective antimicrobial properties, on important bacteria such as methicillin-resistant *S. aureus* (MRSA) [18,19], *Salmonella enterica* [20], and *E. coli* O157 [21], as well as on bacteriophages [22,23] and Norovirus [24]. Copper is used not only for medical applications but is also used for surfaces [16], since it can prevent the spread of pathogens more effectively than stainless steel alone [18] or silver [19].

The antimicrobial mechanisms of copper ions include membrane damage, oxidative stress, and protein/DNA denaturation [25,26,27]. Critically, these are mainly metabolism-independent mechanisms; hence, we hypothesized that copper can kill persister cells. Although copper has already been linked to the induction of persister cells [28,29,30], here, we demonstrate that copper can also effectively kill *E. coli* persister cells.

## 2. Methods

### 2.1. Bacterial Strains, Growth Media and Chemicals

*E. coli* BW25113 was used throughout [31] and was grown at 37 °C, with agitation (250 rpm), in lysogeny both [32]. Cisplatin (cis-diamminodichloroplatinum (II)) stocks (1 mg/mL) were diluted in 0.1 M sodium perchlorate [33], since DMSO inactivates cisplatin [34]. Cupric sulfate stocks (0.5 M) were prepared in water. Both solutions were filter-sterilized.

To determine copper minimum inhibitory concentration (MIC), 10 µL of culture (turbidity at 600 nm of ~1.0) was inoculated into lysogeny broth (LB) medium supplemented with a range of copper concentrations: 0, 120, 240, 480, 960, and 1920 µg/mL. Test tubes (final volume 2 mL) were incubated overnight (approximately 16 h with agitation at 250 rpm), and the MIC was determined as the lowest concentration that prevented bacterial growth by visual inspection. All experiments were conducted with at least two biological replicates.

### 2.2. Persister Cells

Using our previous method [4,5], the persister cell fraction in an *E. coli* culture was increased 10,000-fold, using a rifampicin pre-treatment to induce cells to enter the persister state. To form these persisters, exponentially growing cells at a turbidity of 0.8 at 600 nm were treated with rifampicin for 30 min at 100 µg/mL, harvested by centrifuging at 1600× *g*, and resuspended in ampicillin-supplemented LB (100 µg/mL) to kill non-persister cells for 3 h. Then, the cells were centrifuged and resuspended in LB or NaCl buffer.

### 2.3. ASKA Library Screening for Copper-Resistant Mutants

*E. coli* electro-competent cells (50 µL) were electroporated with 1 µL of pooled plasmids from a complete set of *E. coli* K-12 ORF archive (ASKA) (200 ohms, 25 µF, 1.5 kV) using a 0.1 cm electroporation cuvette. LB medium (900 µL) was added and after 1 h of growth (250 rpm), 990 µL of bacterial suspension was inoculated into 25 mL of LB (no antibiotics added), and cells were grown to a turbidity of approximately 0.5 at 600 nm (250 rpm). Chloramphenicol was added (30 µg/mL), and the cells grown for 1 h. After that, 25 µL of this culture were re-inoculated in LB medium supplemented with cupric sulfate (1 × MIC, 960 µg/mL), with or without IPTG (0.5 mM), and grown overnight (250 rpm). 

### 2.4. KEIO Library Screening for Copper-Resistant Mutants

We used two different approaches to identify copper-resistant strains: LB supplemented with cupric sulfate (1 × MIC, 960 µg/mL) and cupric sulfate in NaCl 0.85% buffer. For both methods, 250 µL of the pooled KEIO mutant library was inoculated into 25 mL of LB and grown to a turbidity of approximately 0.8 (250 rpm) to let the cells revive. Cells (25 µL) were added to LB supplemented with cupric sulfate (960 µg/mL, 1 × MIC in LB), and kept overnight (250 rpm) or 5 mL of the revived cells were centrifuged and washed once in 0.85% NaCl buffer and cupric sulfate at approximately 1/6 × MIC (160 µg/mL, from 1 × MIC in LB of 960 µg/mL). This bacterial suspension was incubated for 2 h without shaking at 30 °C.

### 2.5. Copper Killing Assays

For cells in LB, *E. coli* exponential and persister cultures (5 mL) were centrifuged, resuspended in 5 mL containing 1 × MIC of cupric sulfate (960 µg/mL), and shaken in 125 mL Erlenmeyer flasks; then, the aliquots were used for serial dilutions. Colony-forming units (CFU) were determined after 3 h and 18 h. For cells in NaCl buffer, the cultures were centrifuged, washed 2 × in NaCl 0.85%, and resuspended in this same buffer. To each 2 mL of this bacterial suspension, cupric sulfate was added (32 µg/mL and 160 µg/mL: final concentrations), and cells were incubated without shaking at 30 °C for 1 h.

### 2.6. Effect of Oxygen on Copper Activity

Cells were grown to a turbidity of approximately 0.8 and resuspended in 0.85% NaCl with cupric sulfate (160 µg/mL). Cultures were incubated without shaking at 30 °C for 1 h in opened vials (aerobic) and in an anaerobic chamber (Coy Laboratories). Aliquots were serially diluted and plated on LB–agar plates, which were incubated under aerobic and anaerobic conditions. 

### 2.7. Membrane Integrity Assessment by Live/Dead Microscopy

To determine whether copper ions damage the persister cell membrane, we performed a Live/Dead assay. Persister cells (5 mL) were resuspended in 0.85% NaCl supplemented with cupric sulfate (160 µg/mL final). The bacterial suspensions were incubated for 30 and 60 min at 30 °C incubator, without shaking, and washed twice in PBS buffer before the addition of propidium iodide (PI) (Invitrogen P3566) and SYTO 9 (Invitrogen, L7012), reaching final concentrations of 60 µM and 20 µM, respectively. After a 15 min-incubation (room temperature, light-protected), cells were observed under a Zeiss fluorescence microscope (approximate excitation/emission for PI is 490/635 nm, and for SYTO9, it is 485/500 nm). Pictures taken with different filters were merged using ZEN software. As a control, heat-treated *E. coli* cells were used to confirm the PI staining of dead cells.

### 2.8. Membrane Integrity Assessment by Lysis Assays

To determine if copper ions cause cell lysis, cells were grown to a turbidity of 0.8, washed twice using NaCl 0.85% (3500× *g* for 10 min), and the pellet was resuspended in PBS buffer. For the 100% lysed positive control, a 1 mL aliquot was sonicated (20 s, 3 W, 3 cycles, Sonic Dismembrator 60), centrifuged, and the supernatant used as a positive control (which we call “sonicated”). For the copper-treated cultures (5 mL), cupric sulfate was added (160 µg/mL) and vials were incubated for 1 h at 30 °C; then, they were centrifuged (6500× *g*, 4 °C, 15 min) and the supernatants were collected. Protein detection was determined using BCA protein assay kit (Pierce, Prod#23227), following the manufacturer’s instructions. 

### 2.9. Copper Versus Cisplatin for Killing Persisters

Persister cells were resuspended in LB supplemented with cupric sulfate (1920 µg/mL, 2 × MIC), cisplatin (200 µg/mL, 2 × MIC), or the solvent control for cisplatin, sodium perchlorate (0.1 M), and incubated at 250 rpm. Aliquots for CFU assessment were obtained by serial dilution and colony counting at 0 min, 15 min, 30 min, and 60 min.

### 2.10. Statistical Analysis

All experiments were carried out with at least two biological replicates. Median values were obtained for each time-point assessed, and the standard deviation was calculated. For statistical significance analysis, we used a *t*-test (bicaudal, type 1) versus the control conditions and considered a minimum of 0.05 (*) for the *p*-values.

## 3. Results

### 3.1. Copper-Related Proteins in E. coli K-12

Gram-negative strains utilize the *cue* system (Cu efflux) [35] and the *cus* system (Cu sensing) [36] for copper detoxification. These systems are chromosomally encoded and are complimentary in function. CopA (*cue* system) is involved in cytoplasm detoxification, while the *cus* system is involved in periplasm detoxification [35,37]. Under anaerobiosis, CueO (periplasmic copper detoxification) has its activity impaired, but *cus* system proteins remain functional, removing copper ions from the periplasm [35,37] (Table 1). Under strong selective pressure (i.e., in copper-rich environments), bacteria may also harbor additional plasmid-borne genetic elements that improve copper tolerance (usually *pco* genes) and enhance the ability of the bacterium to efflux copper ions [35]. Our reference strain, *E. coli* BW25113, contains the expected main systems for copper regulation (*cus* and *cue*) and is devoid of the plasmid-encoded *pco* system (Table 1). Therefore, it is a suitable model for copper persistence for *E. coli* and other Gram-negative bacteria. 

### 3.2. Cooper Kills Both Exponential and Persister E. coli Cells

The MIC of copper for *E. coli* exponential cells was determined to be 960 µg/mL in lysogeny broth (LB) after overnight incubation. Persister cells were formed so that they were the dominant population following the previously published methodology [4,5] that increases persister cells by 10,000-fold; this method uses a rifampicin pre-treatment for 30 min to suppress protein synthesis, and a subsequent ampicillin treatment for 3 h to kill non-persister cells. Our method of generating persister cells has been validated eight ways [38] and utilized by 11 independent labs to date [4,39,40,41,42,43,44,45,46,47,48].

**Figure 1:**
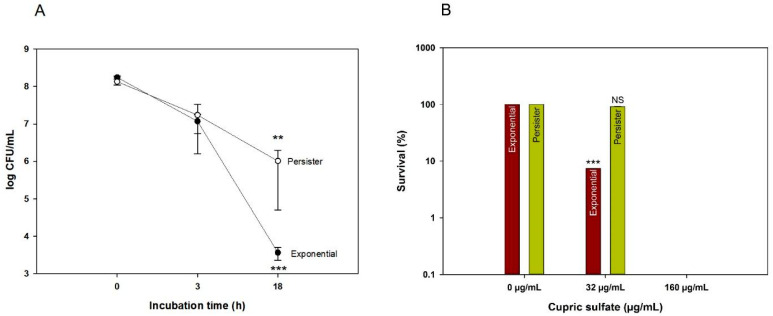
Exponential and persister cell viability with copper in rich medium and in buffer. (**A**) Both exponential and persister cells were incubated for 18 h with copper (960 µg/mL cupric sulfate, 1 × minimum inhibitory concentration (MIC)) in lysogeny broth (LB). Error bars indicate standard deviation. (**B**) Survival under copper stress in 0.85% NaCl buffer after 1 h. Since we could not recover viable cells after incubating with 1 × MIC (160 µg/mL), a ^1^/_5_ concentration was used in buffer (32 µg/mL). Relative survival obtained after copper was added to the bacterial suspension in NaCl buffer. The symbols *** (*p* < 0.001) and ** (*p* < 0.05) indicate significant differences. NS indicates that non-significant difference was found.

Using the MIC for exponentially growing cells, we investigated the ability of copper ions to kill both exponential and persister cells in rich medium (LB) with 1 × MIC cupric sulfate (960 µg/mL). After 18 h of incubation, the number of viable exponentially grown cells was reduced 1000-fold more than the persister cells (Figure 1A); hence, the exponentially grown cells were more susceptible to copper killing. However, the number of viable cells in both populations continually decreased without a plateau, indicating that copper continues to kill both kinds of cells, although it kills the persister cells less rapidly.

Rich medium is not representative of the conditions bacteria face in natural environments, which are predominantly oligotrophic [49]. In addition, copper ions could be complexed by proteins and/or other molecules present in rich media, which could give us less accurate results [50]. In order to evaluate the effect of copper without these artifacts, both persister and exponential cell cultures were washed with 0.85% NaCl buffer before being challenged with cupric sulfate in NaCl buffer. Our first attempts using the LB-obtained MIC of 960 µg/mL resulted in complete cell death, even after short incubation periods of less than one hour (data not shown). Using a lower concentration (32 µg/mL cupric sulfate) and 1 h of incubation, a significant reduction was found in the number of viable exponential cells, in comparison to cells without cupric sulfate, whereas there was no effect on the persister cells (Figure 1B). At higher concentrations (160 µg/mL cupric sulfate), both exponential and persister cells were eradicated (Figure 1B). These results not only corroborate that exponential cells are killed faster than persisters (as shown previously in LB, Figure 1A), but they also show that persisters can be completely eliminated by cupric sulfate.

### 3.3. Copper Ions Damage the E. coli Persister Cell Membrane

Since different mechanisms for copper killing have been suggested [27], including cell lysis [51], we investigated whether cell lysis was occurring with *E. coli* by assessing its membrane status after cupric sulfate treatment via Live/Dead staining. Live/Dead staining uses the membrane-impermeant DNA intercalator propidium iodide and the membrane-permeant DNA intercalator SYTO9 to determine the extent of cell envelope disruption; i.e., cells with membrane damage are stained red, and all cells are stained green by SYTO9. 

**Figure 2:**
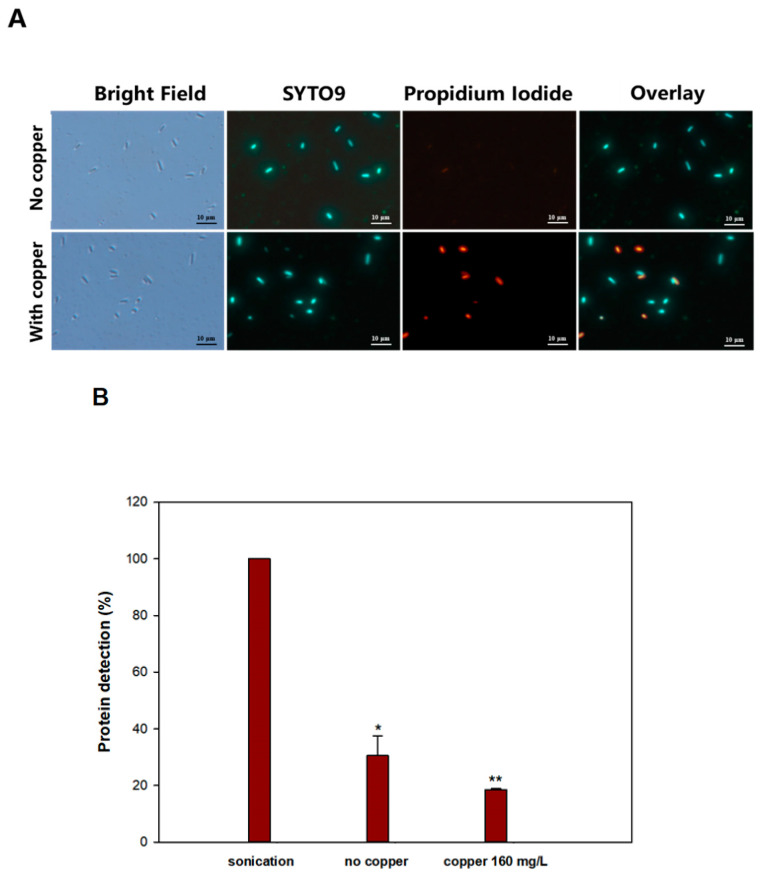
Membrane integrity and cell lysis of persister cells with copper. (**A**) Persister cells were suspended in 0.85% NaCl buffer supplemented with cupric sulfate (160 µg/mL) for 1 h, then treated with Live/Dead staining. Scale bar is 10 µM. (**B**) Persister cells were treated with copper (160 µg/mL) or 1 × PBS buffer (“no copper”) and protein content was quantified and compared to the positive control of completely-lysed cells via sonication (“sonicated”) * (*p* < 0.05); ** (*p* < 0.01).

The microscopy results show that after 160 µg/mL of cupric sulfate in NaCl buffer for 1 h, 58% ± 8% of persister cells have damaged membranes (Figure 2A). The remaining green-stained cells for the copper-treated cultures are also dead, since no surviving cells were recovered with these conditions (Figure 1B). Therefore, membrane damage occurs to a significant extent due to the copper treatment of persister cells, and copper ions might also impair some internal biochemical pathways, leading to cell death.

In addition, we hypothesized that if membrane damage was the leading cause of death by copper ions, we would be able to detect an increase in protein content outside of cells. Therefore, supernatants of the suspensions of persister cells after copper treatment (160 µg/mL of cupric sulfate in NaCl buffer for 1 h) were analyzed, and we found no additional extracellular protein after copper treatment (Figure 2B); hence, we did not find evidence of cell lysis, only evidence of membrane damage.

### 3.4. Copper Ions Kill More Effectively Anaerobically

**Figure 3:**
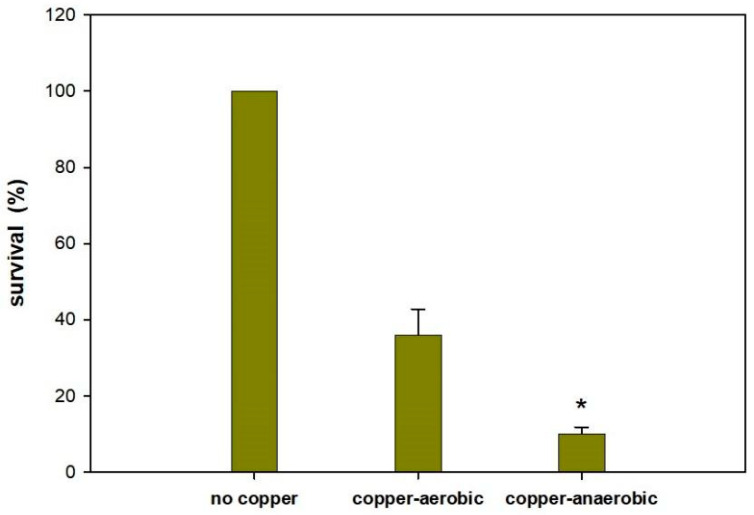
Exponentially growing cells with copper under aerobic and anaerobic conditions. Bacterial suspensions in 0.85% NaCl buffer supplemented or not with 32 mg/mL cupric sulfate for 60 min at 30 °C. A “no copper” control was used for each condition for comparison; i.e., for both anaerobic and aerobic conditions and is listed as 100%. The symbol * (*p* < 0.05) indicates the significant difference compared to the control (“no copper”).

Since copper ions has been reported to kill *E. coli* cells more effectively under anaerobic conditions [51,52], we tested the effect of oxygen on cupric sulfate at 32 µg/mL in NaCl for 1 h for exponentially grown cells. As expected, we found a greater killing of exponential cells in the absence of oxygen (91% ± 2% versus 64% ± 6%, Figure 3).

### 3.5. Absence of Copper Resistance Mutants

Realizing genetic resistance could limit the use of copper as an effective antimicrobial, we assessed the likelihood of copper resistance using pooled libraries [12] of the 3985 KEIO *E. coli* single-gene knockouts which contains all non-lethal mutations of *E. coli* K-12 [31] as well as pooled libraries [2] of the ASKA plasmid library [53] where all of the 4287 *E. coli* proteins are produced. We reasoned that either a deletion or production of a key protein would allow higher copper tolerance and that this approach could also give us additional clues about the mode of action of copper.

**Figure 4:**
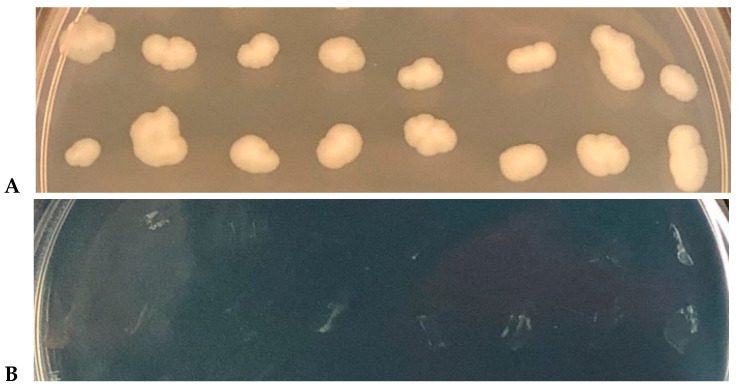
Copper-tolerant mutant strains are unable to withstand 1 × MIC in rich medium. (**A**) Copper tolerant mutants obtained after a 2 h exposition to 160 µg/mL cupric sulfate in NaCl buffer grow normally in LB agar plates after an overnight incubation without copper. (**B**) The same mutants shown in A, but now on a copper-supplemented LB agar plate (1 × MIC, 960 µg/mL). Note that despite being selected for being more tolerant to copper in a short-duration experiment (in NaCl buffer for 2 h), the cells are unable to survive at 1 × MIC when incubated.

For the pooled ASKA strains, after transformation, cells were grown overnight in LB supplemented with 1 × MIC of cupric sulfate (960 µg/mL), with and without 0.5 mM IPTG to induce gene expression. However, no strain capable of growing at 1 × MIC was recovered. For the pooled Keio strains, since 160 µg/mL of cupric sulfate reduces a 10^8^ cell/mL bacterial suspension to zero in 1 h in NaCl buffer (Figure 1B), we exposed the pooled library for 2 h at this condition. Some colonies were recovered using this method, but further testing showed that despite being more tolerant to copper ions during the limited-time exposure (2 h), it did not render these cells able to grow at 1 × MIC (960 µg/mL) during an overnight incubation (Figure 4). Hence, no copper-resistant mutants were obtained.

### 3.6. Copper Ions Are More Effective than Cisplatin for Eradicating Persisters

**Figure 5:**
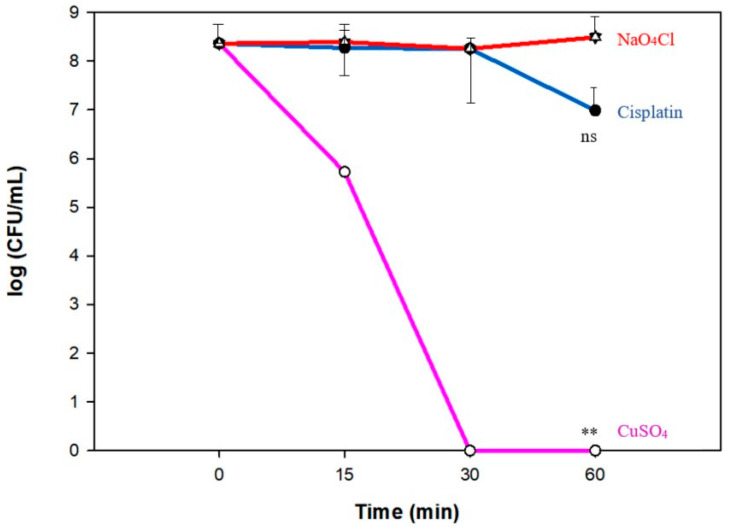
Copper is more effective than cisplatin for killing persister cells. Persister cells were treated with cisplatin (200 µg/mL, 2 × MIC), cupric sulfate (1920 µg/mL, 2 × MIC), or sodium perchlorate (solvent control for cisplatin) for 30 min in LB. Aliquots for CFU assessment were taken at 0, 15, 30, and 60 min. The symbol ** (*p* < 0.01) indicates the significant difference compared to the control (NaO_4_Cl), using all the time-points values for each condition as a matrix to compare with all the time points obtained for the control. “ns” indicates that a non-significant difference was found.

We compared the effectiveness of copper ions to another well-known persister-killing molecule, cisplatin [10], for killing *E. coli* persisters. After 30 min of incubation with 2 × MIC for each of these chemicals in LB, copper ions eradicated the persister cells faster than cisplatin (Figure 5), demonstrating that copper is more effective than cisplatin for eliminating *E. coli* persister cells.

## 4. Discussion

Although copper is an essential element for life [54,55,56], it is toxic [57,58], and many different modes of action have been proposed for this molecule. Besides lipid and DNA oxidation [15], proteins can suffer mismetallation [59], misfolding [60], and the destruction of Fe–S clusters [61] in the presence of copper. Interestingly, the reason for the essentiality and destructive potential of copper is the same: it is highly reactive [54,56]. After the Earth’s atmosphere became oxygenated, the conversion of Cu^+1^ to Cu^+2^ increased the availability of this metal availability for biocatalysis [15,62]. As a result, many respiratory-related proteins need this metal to function properly [63], but its homeostasis needs to be strictly controlled by the cell to avoid its lethality [15,64].

Despite a matter of debate, the formation of reactive oxygen species (ROS) was thought to be one of the main reasons for copper ions’ toxicity [53,61]. Indeed, oxidative stress occurs [61], but it might be the result of downstream effects, after the primary targets are attacked by copper ions [35,52,61]. This has been shown by recent studies that indicate that the primary reason for copper toxicity is the destruction of an Fe–S cluster of proteins, which is an oxygen-independent process that is enhanced under anaerobic conditions [52,62,65]. In our study, we also found that under anaerobic conditions, copper ions were more lethal to exponentially grown *E. coli* cells (Figure 3), and our attempts to reduce ROS from copper by using glutathione were not successful (data not shown). 

Other modes of action for copper have also been proposed, including contact killing by cell envelope-associated damage [27,66]. In our study, we found clear evidence of membrane damage (Figure 2A) but not lysis, since no extra cellular protein (Figure 2B) was found. Regardless of its exact killing mechanism, copper ions are potent antimicrobial molecules. In fact, macrophages utilize copper ions to kill microbes [67,68] and copper deficiency in humans and animals is linked to an increase in infection [69]. 

Unlike many antibiotics, copper resistance is unlikely to occur spontaneously [70], requiring the acquisition of plasmid-based operons to enhance copper homeostasis [71]. This may explain our unsuccessful attempts to recover copper-resistant mutants using both KEIO and ASKA libraries; however, more testing is required using longer times for mutants to occur and strains that are more medically relevant. Genomic analysis performed on *E coli* BW25113 showed that it has no *pco* operon, although copper homeostasis genes were fully present. Additionally, many medically relevant *E. coli* strains are susceptible to copper [21], and even in the presence of supplemental copper-resistance genes, most bacteria die on cupric surfaces, although they may need more contact time for this to happen [72,73,74].

## 5. Conclusions

Critically, one of the main results of this work is that due to the metabolism-independent membrane damage caused by copper ions, we verified that it is capable of killing *E. coli* persister cells (Figure 1). Although the medical use of copper requires steps to limit its toxicity, our results suggest new therapeutic alternatives to control persister cells, as well as suggest a continued use of copper surfaces to kill bacteria. For example, copper-coated surfaces in hospitals could decrease the occurrence of healthcare-associated infections, which can reach rates as high as 6% of acute care inpatients [75]. In addition to surfaces, many other uses of copper ions could be envisioned [76], especially because there are other important sources of contamination inoculum in hospitals, besides hand-touched surfaces, such as white coats [76,77,78]. To our knowledge, this is the first study to show that cupric compounds can effectively kill persister cells. Hence, in addition to the long-known antimicrobial properties of copper materials, its use could be expanded to include dormant cells, helping in the ever-increasing need to fight pathogens.

## Figures and Tables

**Table 1:** Gram-negative copper homeostasis/resistance systems (Bondarczuk and Piotrowska-Seget, 2013) identified in *E. coli* BW25113.

System	Gene	Comments
***cue* system** **“Cu efflux”**	*cueR*	copper-responsive metalloregulatory protein
	*cueO*	multi-copper oxidase (periplasmic copper detoxification)
	*copA*	soft metal ion-translocating ATPases (extrudes the excess copper from the cytoplasm into the periplasm)
***cus* system** **“Cu sensing”**	*cusC*	efflux system spanning the entire cell envelope plus periplasmic copper detoxification
	*cusB*	
	*cusF*	
	*cusA*	
	*cusR*	
***pco* system** **“plasmid-borne copper resistance”**	*pcoABCDRSE*	*pco* system requires CopA activity to confer resistance
